# Melanocortin receptor agonists MCR_1‐5_ protect photoreceptors from high‐glucose damage and restore antioxidant enzymes in primary retinal cell culture

**DOI:** 10.1111/jcmm.13036

**Published:** 2016-12-20

**Authors:** Rosa Maisto, Carlo Gesualdo, Maria Consiglia Trotta, Paolo Grieco, Francesco Testa, Francesca Simonelli, Jorge Miquel Barcia, Michele D'Amico, Clara Di Filippo, Settimio Rossi

**Affiliations:** ^1^ Department of Experimental MedicineDivision of PharmacologySecond University of NaplesNaplesItaly; ^2^ Multidisciplinary Department of Medical‐Surgical and Dental SpecialitiesSecond University of NaplesNaplesItaly; ^3^ Pharmacy DepartmentUniversity of Naples Federico IINaplesItaly; ^4^ School of MedicineUniversity of ValenciaValenciaSpain

**Keywords:** hyperglycaemia, oxidative stress, primary retinal cell cultures, photoreceptors, melanocortin receptor agonists

## Abstract

Retinal photoreceptors are particularly vulnerable to local high‐glucose concentrations. Oxidative stress is a risk factor for diabetic retinopathy development. Melanocortin receptors represent a family of G‐protein‐coupled receptors classified in five subtypes and are expressed in retina. Our previous data indicate that subtypes 1 and 5 receptor agonists exert a protective role on experimental diabetic retinopathy. This study focuses on their role in primary retinal cell cultures in high‐glucose concentrations. After eye enucleation from wild‐type male C57BL/6 mice, retinal cells were isolated, plated in high‐glucose concentration and treated with melanocortin receptors 1 and 5 agonists and antagonists. Immunocytochemical and biochemical analysis showed that treatment with melanocortin receptors 1 and 5 agonists reduced anti‐inflammatory cytokines and chemokines and enhanced manganese superoxide dismutase and glutathione peroxidase levels, preserving photoreceptor integrity. According with these evidences, we propose a major role of melanocortin receptors 1 and 5 on primary retinal cell response against high glucose or oxidative insults.

## Introduction

Hyperglycaemia is one of the most common complications of diabetes leading to vision impairment worldwide 1, 2. Hyperglycaemia is also accompanied by oxidative misbalance. Briefly, oxidative stress can be considered as a pro‐oxidant over drive *versus* the antioxidant ones 3. Several reports have focused on the relevance of oxidative stress for diabetes outcome. In fact, the use of co‐adjuvant antioxidant therapies may result helpful for the management of this disease 4, 5, 6.

Among the antioxidant enzymes, manganese superoxide dismutase (MnSOD) and glutathione peroxidase (GPx) play a key role on the antioxidant cell machinery. GPx and MnSOD are crucial for oxidative balance on neural tissue including retina 4, 7. More concretely, it has been reported that catalase, GPx and MnSOD genes are significantly reduced in patients with diabetic retinopathy 8.

Retina is a complex neural cell layer lining the inner surface of the eye involved in processing of visual stimuli 9. Several cell types are included on retina for example amacrine cells, Müller cells, ganglion cells and photoreceptors among others. It has been demonstrated that retinal photoreceptors are particularly vulnerable to local high‐glucose concentrations 10 and oxidative stress is a risk factor for diabetic retinopathy development 11, and retinal photoreceptor alterations may play an important role in the progression of diabetic retinopathy 12.

Melanocortin receptors (MCR) represent a family of G‐protein‐coupled receptors classified in five subtypes (MCR_1‐5_) 13, 14, 15. MCR are expressed in several tissues including retina 9, 16, and they can be activated or inhibited by either agonists as α‐melanocyte‐stimulating hormone (α‐MSH) or antagonists as agouti‐related protein 17. MCR agonists as α‐MSH preserve from rat dry‐eye alterations *via* protein kinase A–cAMP response element‐binding protein (PKA‐CREB) and extracellular signal‐regulated kinases–protein kinase B (ERK‐AKT) pathways 18 and more concretely, it protects retinal pigment epithelium from oxidative stress by activating the melanocortin receptor 1–protein kinase B–mammalian target of rapamycin complex 1 (MCR_1_‐AKT‐mTOR) pathway 19. Fitting with this, previous data from our laboratory indicate that MCR_1_ and MCR_5_ receptor agonists exert a protective role on experimental diabetic retinopathy by modulating the pattern of cytokine and chemokine expression 13.

Following this previous data from our laboratory, here we would like to test whether the protective role of MC_1,5_ receptors is exerted on some proper structures of the retina such as the photoreceptors. Thus, a study was undertaken on a primary retina‐cell culture stimulated with high‐glucose concentrations and with MCR_1,5_ agonists were used under high‐glucose conditions to delve into the protective role of MCR agonists on photoreceptors, through evolution of the expression and levels of two specific photoreceptor markers as opsin and recoverin.

## Material and methods

BMS‐470539 and PG‐901 were used as MCR_1_ and MCR_5_ agonist, respectively, although PG901 also binds with antagonistic activity MC3R and MC4R 20, 21. Compounds were supplied by Professor Grieco (Pharmacy Department, University of Naples Federico II).

### Animals

All the experimental procedures were performed according to the Second University of Naples guidelines of the Ethic Committee for animal experiments. Three‐week‐old male C57BL/6 mice (18–22 g) (Harlan, Milan, Italy) were housed in standard cages (*n* = 10 per cage) with a cycle of 12 hrs light (7 a.m. to 7 p.m.) and 12 hrs dark, humidity and temperature automatically controlled to 60% and 21 ± 1°C, respectively.

### Retinal cell cultures

Retinal cell cultures were obtained according to Santiago *et al*. 2 with some modifications. Briefly, mice (*n* = 10) were anesthetized by intraperitoneal injection of ketamine/medetomidine (ketamine 100 mg/kg and medetomidine 0.25 mg/kg). After eye enucleation, retina was dissected under sterile conditions using the enzymes trypsin and collagenase A 22. After dissociation, the cells were collected by centrifugation and resuspended in Eagle's minimum essential medium (MEM) supplemented with 26 mM NaHCO_3_, 25 mM HEPES, 10% heat‐inactivated foetal bovine serum, penicillin (100 U/ml) and streptomycin (100 μg/ml). The cells were maintained in humidified atmosphere of 5% CO_2_ air at 37°C. The cells were plated at a density of 2.0 × 10^6^ cells per cm^2^ on 24‐well plates or 35 mm Petri dishes, coated with poly‐d‐lysine (0.1 mg/ml; Sigma‐Aldrich, St Louis, MO, USA). Two days after, cells were incubated for 20 days with high‐glucose concentration 25 mM d‐glucose (high glucose) or 5 mM d‐glucose (control) 11. After this, retinal cell cultures were treated for 24 hrs with MC‐r agonists PG‐901 (MC_5_ agonists, 10^−10^ M); (BMS‐470539, 10^−5^ M) 23. Each treatment was repeated three times.

### Immunocytochemistry

Cells cultured in glass coverslip were fixed with 4% paraformaldehyde in PBS pH 7.4 for 10 min. at room temperature. After fixation, the cultures were washed with PBS and incubated 1 hr with blocking solution 5% BSA serum (Sigma‐Aldrich) 0.05% Tween in PBS, and then incubated overnight with monoclonal anti‐opsin (1:1000; Sigma‐Aldrich) and anti‐recoverin (1:1000; Abcam, Cambridge, UK) antibodies. Alexa Fluor^®^ 488 (Jackson Laboratory, West Baltimore Pike, West Grove, PA, USA)‐conjugated goat polyclonal antibody (1:1000) was used as secondary for opsin detection. Cy3‐conjugated goat polyclonal anti‐rabbit (Jackson Laboratories; 1:400) was used as secondary for recoverin detection. Nuclei were counterstained by DAPI. Quantification of fluorescence intensity was determined by LEICA software (Milan, Italy). The method used by Alessio *et al*. 24 was applied to calculate the percentage of positive cells in each microscope field. This was calculated by the number of green or red (opsin or recoverin) positive cells of 400 cells in six different microscope fields according to the previous method 24.

### Western blotting

Western blotting was performed on retinal cell lysates obtained following the protocol described by Baptista *et al*., 10. Briefly, cells were washed with ice‐cold phosphate‐buffered saline (PBS, in mM: 137 NaCl, 2.7 KCl, 10 Na_2_HPO_4_, 1.8 KH_2_PO_4_, pH 7.4, at 4°C) and lysed with RIPA buffer (50 mM Tris–HCl, pH 7.4, 150 mM NaCl, 5 mM EDTA, 1% Triton X‐100, 0.5% DOC, 0.1% SDS, 1 mM DTT) supplemented with complete miniprotease inhibitor cocktail tablets and phosphatase inhibitors (10 mM NaF and 1 mM Na_3_VO_4_). Lysates were incubated on ice for 30 min. and centrifuged at 16,000 × g for 10 min. at 4C°. The protein concentrations were determinate as described by Bradford (1976). The primary polyclonal antibodies used are anti‐manganese superoxide dismutase MnSOD (dilution 1:200; Millipore, Merck, Milan, Italy) and anti‐glutathione peroxidase (GPx) (dilution 1:200; Abcam, UK). Anti‐b‐actin was used as loading control, with an enhanced chemiluminescence detection reagent (ECL). Protein bands were quantified by densitometry performed with a Bio‐Rad ChemiDoc MP Imaging system. Secondary antibodies used were anti‐mouse and anti‐rabbit (dilution 1:1000; Santa Cruz Biotech, CA, USA).

### RT‐PCR

Total RNA was extracted using RNeasy Plus Mini Kit (Qiagen, West Sussex, UK), according to the manufacturer's instructions. Contaminating DNA was removed from RNA preparations performed with the Ambion^®^ Thurbo DNA‐free system (Life Technologies, Waltham, MA, United States) using manufacturer's instructions. The concentration and purity of the RNA were then analysed using the Nanodrop ND‐1000 (NanoDrop Technologies, Wilmington, DE, USA). Complementary DNA (cDNA) was obtained by reverse transcription (RT) of 1 μg of total DNase‐treated RNA, with the Superscript III reverse transcriptase system (Invitrogen, Carlsbad, CA, USA) and oligo(dT)_15_ as a primer following manufacturer's protocol. Real‐time PCR was performed with Read Mix PCR Master Mix (ThermoScientific, Waltham, MA, United States) and the following amplification profile: 95°C for 2 min.; 35 cycles ‐ 94°C for 30 sec., 55°C for 35 sec. and 72°C for 65 sec., followed by final elongation step at 72°C for 5 min. Each 25 μl reaction consisted of 1 μl of diluted cDNA (150 ng/μl RNA), 22.5 μl of 1.1× ReddyMix PCR MasterMix, 1 μl of ddH_2_O and 1 μl of commercially available primer for amplification of mouse MC1R and *MCR*_*5*_ (Qiagen). mRNA data were normalized relative to *GAPDH* and then used to calculate expression levels. Negative controls were either RT without enzyme or PCR without cDNA template. The protocol for the RT‐PCR was performed according to Siniscalco *et al*., 25.

### MCR_1_ and MCR_5_ protein levels

MCR_1_ and MCR_5_ protein levels were determined by a commercial Elisa kit (Biosource, San Diego, CA, USA and Canada), according to manufacturer's protocol.

### Statistical analysis

The results of each experiment are presented as mean ± S.E.M. of the three treatments. Statistical significance was determined using anova followed by Bonferroni's test. For the immunocytochemistry, the mean ± S.E.M. of the percentages was calculated and expressed in graph. Differences were considered significant when *P < 0.05 *versus* high glucose, **P < 0.01 *versus* high glucose and °*P* < 0.01 *versus* control.

## Results

### MCR_1_ and MCR_5_ gene expression and protein in retinal cells cultured in high glucose

**Figure 1:**
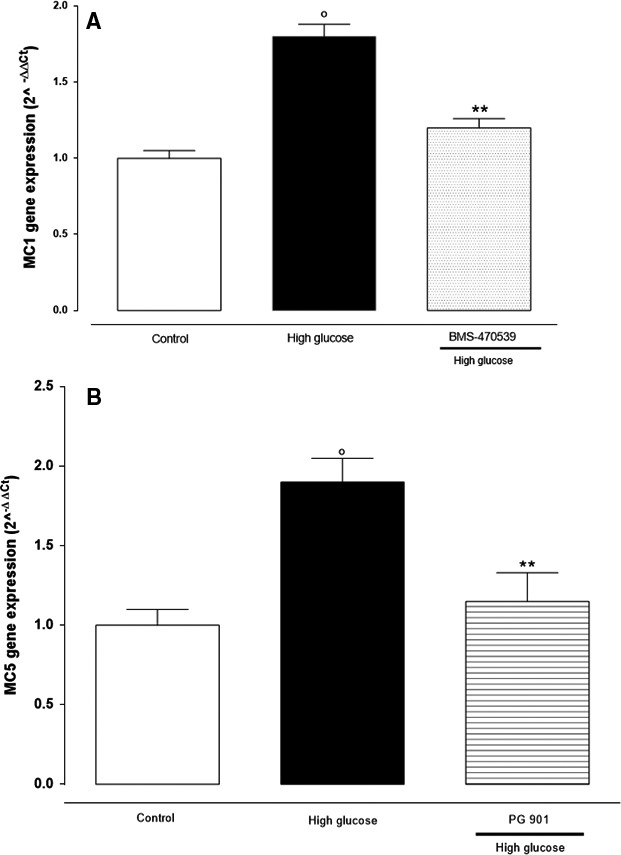
RT‐PCR analysis showed **(A)** MCR_1_ (melanocortin receptors 1) and **(B)** MCR_5_ (melanocortin receptor 5) gene expression in retinal cells cultured in high‐glucose (25 mM) concentration, and in the presence or absence of MCR_1_ agonist BMS‐470539 and MCR_5_ agonist PG‐ 901. The results are reported as the mean ± S.E.M. of *n* = 3 treatments and the significant results expressed as °*P* < 0.01 *versus* control and **P < 0.01 *versus* high glucose.

RT‐PCR showed a significant increase of MCR_1,5_ gene expressions in retinal cells after high‐glucose exposure compared to control cells (*P* < 0.01 *versus* control). In contrast, both MCR_1,5_ genes were significantly reduced (*P* < 0.01 *versus* control) in the presence of the MCR_1,5_ agonists (PG901 and BMS‐470539, respectively) (Fig. 1A and B).

**Figure 2:**
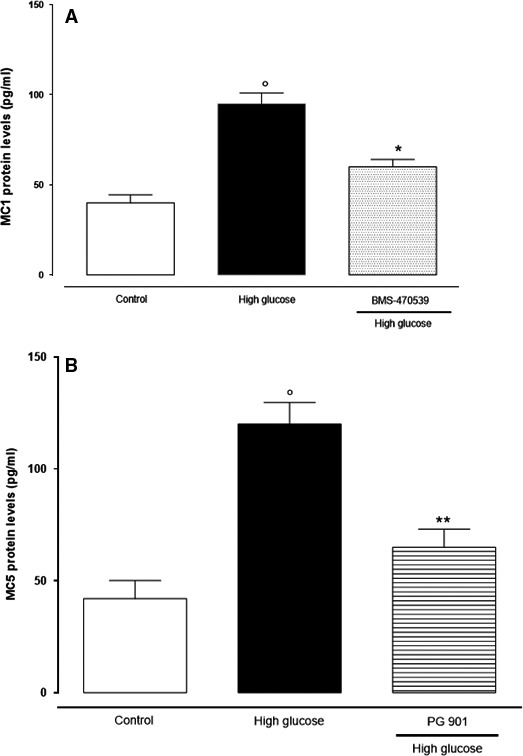
ELISA assay showed high levels of MCR_1,5_ protein in retinal cells cultured in high‐glucose concentration. These were significantly decreased by the treatment with BMS‐470539 and PG‐ 901 (MCR_1_ and MCR_5_ agonists). The results are reported as the mean ± S.E.M. of *n* = 3 treatments and the significant results expressed as °*P* < 0.01 *versus* control and **P < 0.0 *versus* high glucose.

To confirm gene expression data, MCR_1,5_ protein levels were measured by ELISA assay, and fitting with RT‐PCR results, protein levels show the same expression profile. MCR_1,5_ protein levels were significantly increased under high‐glucose conditions. Consistently with RT‐PCR, MCR_1,5_ agonists were able to reduce the high‐glucose‐increased MCR_1,5_ protein levels (Fig. 2A and B).

### Decreased MnSOD and GPx enzyme levels are restored by MCR_1_,_5_ agonists

**Figure 3:**
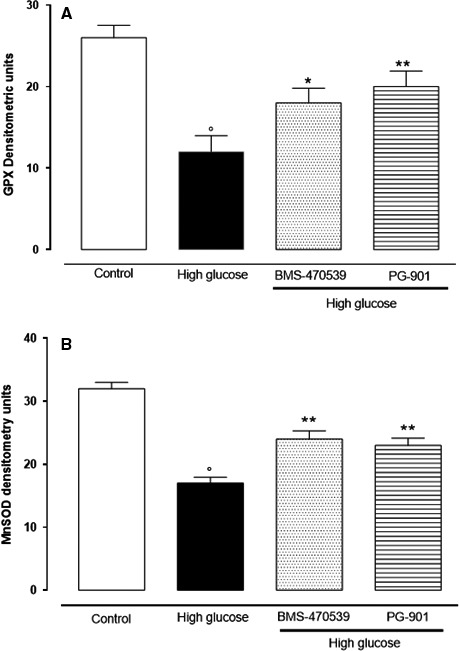
Western blotting analysis showed that high glucose (25 mM) decreases MnSOD and GPx enzyme levels; (**A, B**). Treatment with the compounds BMS‐470539 (MCR_1_ agonist) and P‐901 (MCR_5_ agonist) restored the MnSOD and GPx enzyme levels. (**A, B**). MnSOD: manganese superoxide dismutase; GPx: glutathione peroxidase. The results are reported as the mean ± S.E.M. of *n* = 3 treatments and the significant results expressed as *P < 0.05 *versus* high glucose, **P < 0.01 *versus* high glucose and °*P* < 0.01 *versus* control.

MnSOD and GPx antioxidant enzymes were significantly decreased after high‐glucose exposure compared to normal glucose (control) cultured cells (Fig. 3A and B). Conversely, MnSOD and GPx levels were significantly increased after MCR_1,5_ agonist treatment (Fig. 3A and B).

### Opsin and recoverin cell labelling

**Figure 4:**
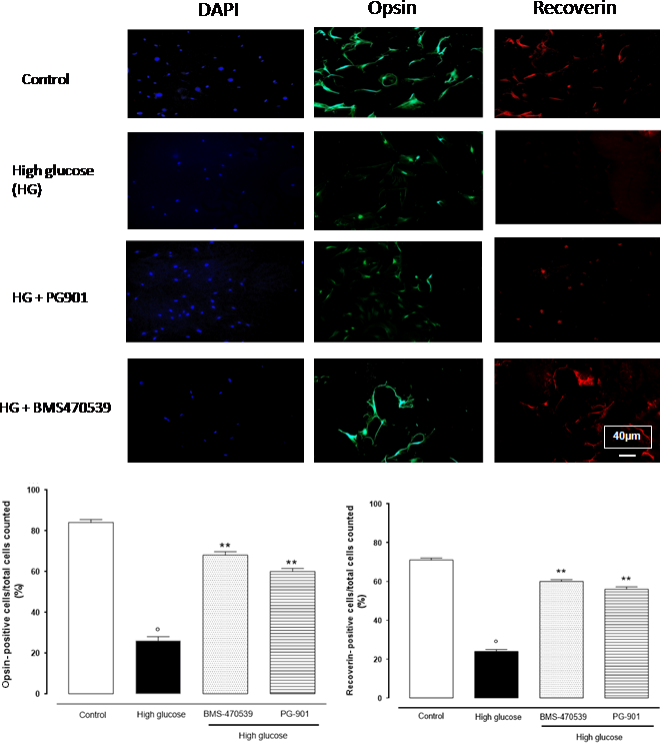
Depicted are representative immunocytochemistries of retinal cells cultured in 5 mM or 25 mM glucose and labelled with opsin, recoverin and 4′,6‐diamidino‐2‐phenylindole (DAPI) antibodies. Cells were treated with BMS‐470539 and PG‐901, and representative microscopic fields for each treatment are shown. Accordingly, the percentage of recoverin and opsin‐positive cells is represented in the graph. The results are expressed as mean ± S.E.M. of the percentages of positive cell/total cell counted in each analysed field for each treatment. The statistical significance was reported as °*P* < 0.01 *versus* control; **P < 0.01 *versus* high glucose. 40× magnification.

Among the different cell types included in the retinal cell culture, the presence of photoreceptors can be recognized by the presence of recoverin and opsin. Under control conditions (5 mM Glucose), photoreceptors exhibit large cytoplasm expansions, and opsin is sparsely distributed along the cytoplasm membrane (Fig. 4). In contrast, photoreceptors exposed to high‐glucose concentration (25 mM) present less opsin labelling (Fig. 4), as evidenced by the percentage of opsin‐positive cell on the total of cells counted. However, the addition of the MC receptor agonists, PG901 and BMS‐470539, and melanocortin receptors 1 and 5 to high‐glucose‐treated photoreceptors presented a pattern of opsin labelling more similar to that shown after control conditions (Fig. 4).

Control recoverin‐positive cells present a red dye with cytoplasm location (Fig. 4). However, high‐glucose‐treated cells present almost null recoverin reactivity (Fig. 4). In contrast, the addition of PG901, or BMS‐470539, resulted in evident pattern of labelling similar to that observed under control conditions (Fig. 4).

Structurally, high‐glucose‐(25 mM) cultured cells appear with abnormal morphology of photoreceptors characterized by stringy, swelled and compressed size, with respect to the control (5 mM). In contrast to this, treatment of high‐glucose‐cultured cells with the compounds BMS‐470539 and PG‐901 improved photoreceptors morphology that indeed appear less distorted, more regular and more similar to the control cells (Fig. 4).

## Discussion

The present study shows that murine primary retinal cells exposed to a high‐glucose medium express a damaged photoreceptors phenotype. This demonstrated by a morphological assessment and by a decrease of two markers of cell membranes and photoreceptors integrity, the opsin and recoverin onto the cell surface.

It is well known that high glucose in diabetes is an independent risk factor for several vascular and non‐vascular diseases 26, and promotes direct cellular alterations by inducing a stress response independently of the diabetic condition 26, 27, 28. At level of the retina, a persistent hyperglycaemia leads derangement of retinal vessels and retinal structure causing retinopathy 13. Several previous studies indicated different pathways and pattern of mediators as responsible of this damage, including oxidative stress and inhibition of antioxidant enzyme gene expression 4, 8, 29. They do not describe, however, the role of melanocortin peptides and their receptors in this mechanism. Endogenous melanocortins are peptides that control many physiological and pathological processes through the activity of different 7‐transmembrane G‐protein‐coupled receptors called MCR_1‐5_ 13. These MCR, probably due to their role on skin cancer, skin‐related diseases or even obesity 30, MCR, have attracted attention of many researchers on the last two decades, from 75 results in 1998 to 270 results in 2015 (PubMed). Indeed, beyond melanocyte regulation, MCR are related to other cell‐signalling pathways such as the leucocytes activation, the promotion of inflammation resolution and the consequent tissue protection 13. Moreover, it has been shown that α‐MSH or other MCR agonists has immunosuppressive activity in experimental uveitis 25, 31 and also protects retinal endothelial cells from oxidative‐induced damage 32. Fitting with this knowledge, in an initial study, we described for the first time that MCR_1,5_ agonists help diabetic retinopathy by concretely protecting retinal vascular network 13, 32 in a murine model, through the inhibition of the local inflammatory and immune responses 13. To these pioneering results have been added now the new data of an antioxidant and defensive response of the retinal cells following activation of MCR_1,5_. Particularly, here we show that MCR_1,5_ agonists promote a protective response on photoreceptors of high‐glucose‐cultured primary retinal cells by preserving their structure from the abnormal morphology and cytoplasm swelling induced by high glucose. High glucose also promotes an evident MCR_1,5_ overexpression in these cells, and MCR_1,5_ agonists normalize this increase. From the biochemical point of view, the activation of the MCR_1,5_ was accompanied by restoring of the levels of both GPx and MnSOD enzymes, impaired by high‐glucose exposure 13. Noteworthy, impaired antioxidant enzymes by high‐glucose results in a high accumulation of hydrogen peroxide (H_2_O_2_) and superoxide (O_2_^•−^), and a reduced nitric oxide (NO) bioavailability 33, thus driving retinal cells towards derangement.

Photoreceptors are case sensitive to high‐glucose conditions 10 playing a pivotal role on diabetic retinopathy 12. In fact, photoreceptor cell membranes are particularly rich in polyunsaturated fatty acids and extremely vulnerable to oxidative damage being the major site of superoxide generation in diabetes 34. As MCR_1,5_ modulate the nuclear transcription of the cAMP response element‐binding protein (CREB) 17, 35 and CREB as a redox‐regulated pathway modulating MnSOD transcription 36, a possible theoretical frame, supporting this proposal, is that high‐glucose exposure overexpresses MCR_1,5_ and the addition of MCR_1,5_ agonists lead to cAMP‐PKA‐CREB, increasing MnSOD transcription.

Beyond this and in view of the present results, we cautiously propose a major role of MCR_1,5_ on cell response against high glucose or other oxidative insults. On another note, previous finding have shown that agonism with MTII (dual MCR_3,4_ agonist) or antagonism with SHU9119 (dual MCR_3,4_ antagonist) did not affect phenotype of the retina 13. Future research must be focused on the MCR_1,5_ overexpression significance and how oxidative stress lead to this MCR_1,5_ overexpression. A better knowledge on the molecular basis of MCR_1,5_ system would be of interest for developing MCR agonist‐based therapies against diabetic complications, especially if the research will prepare more selective and powerful compounds towards the MCR_5_ than the PG‐901 compound used in the present study.

## Conflict of interest

The authors declare that there is no conflict of interests regarding the publication of this study.
